# Recent studies on resveratrol and its biological and pharmacological activity

**DOI:** 10.17179/excli2017-253

**Published:** 2017-04-27

**Authors:** Yong Joo Kim, Sun Ok Chung, Jae Kwang Kim, Sang Un Park

**Affiliations:** 1 Department of Biosystems Machinery Engineering, Chungnam National University, 99 Daehak-ro, Yuseong-gu, Daejeon, 34134, Korea; 2 Department of Crop Science, Chungnam National University, 99 Daehak-ro, Yuseong-gu, Daejeon, 34134, Korea; 3 Division of Life Sciences and Convergence Research Center for Insect Vectors, Incheon National University, Incheon 22012, Korea

## ⁯

Dear Editor,

Resveratrol (3,5,4′-trihydroxy-trans-stilbene) is a stilbenoid and polyphenolic compound. It is found naturally, especially in plants sources such as peanuts, grapes, and some berries (Diaz-Gerevini et al., 2016[9]). In plants, resveratrol is produced via the phenylpropanoid pathway. It is derived from *p*-coumaric acid, which is an intermediate formed during lignin production. The 4-coumarate:coenzyme A (CoA) ligase converts *p*-coumaric acid to coumaroyl-CoA, along with three units of malonyl-CoA, which are then condensed to form resveratrol by resveratrol synthase or stilbene synthase (Deng et al., 2016[8]; Zhang et al., 2015[55]; Zheng et al., 2015[57]; Kim et al., 2013[21]).

Resveratrol is the most widely studied plant-derived polyphenol. Several studies have been reported on its numerous biological and pharmacological effects. These include neuroprotective (Su et al., 2016[45]), antiobesity (Chang et al., 2016[5]), antiviral (Abba et al., 2015[1]), hepatoprotective (Faghihzadeh et al., 2015[11]), anti-inflammatory (Liu et al., 2015[27]), cardioprotective (Raj et al., 2015[40]), anticancer (Kumar et al., 2015[22]), anti-atherogenic (Riccioni et al., 2015[41]), antidiabetic (Szkudelski and Szkudelska, 2015[46]), and antioxidant (Yun et al., 2014[53]) activities. Resveratrol shows a wide range of biological activities and health benefits in humans, which makes it a beneficial substance for use in the pharmaceutical, food, and cosmetic industries. We present a review of the most recent studies on the benefits of resveratrol, especially its biological and pharmacological activities (Table 1(Tab. 1)). (References in Table 1: Zhai et al., 2016[54]; Hyatt et al., 2016[14]; Tsang et al., 2016[49]; Rai et al., 2016[39]; Li et al., 2016[25]; Jin et al., 2016[18]; Abdel-Wahab et al., 2016[2]; Kim et al., 2016[20]; Su et al., 2016[45]; Lai et al., 2016[23]; Hu et al., 2016[13]; Monserrat Hernández-Hernández et al., 2016[32]; Natalin et al., 2016[33]; Chang et al., 2016[5]; Polley et al., 2016[37]; Bedada et al., 2016[4]; Shah et al., 2016[43]; Khazaei et al., 2016[19]; Li et al., 2016[26]; Das et al., 2015[7]; Wang et al., 2015[50]; Meng et al., 2015[31]; Tan et al., 2016[47]; Sin et al., 2015[44]; Ishikawa et al., 2015[15]; Lu and Wang, 2015[28]; Ji et al., 2015[17]; Rabassa et al., 2015[38]; Pektaş et al., 2015[35]; Jang et al., 2015[16]; Meftahi et al., 2015[30]; Dong et al., 2015[10]; Pereira et al., 2015[36]; Schroeter et al., 2015[42]; Lee et al., 2015[24]; Yaylali et al., 2015[52]; Arslan et al., 2015[3]; Trung et al., 2015[48]; Ortega and Duleba, 2015[34]; Riccioni et al., 2015[41]; Mastromarino et al., 2015[29]; Yao et al., 2015[51]; Cigremis et al., 2015[6]; Zhao et al., 2015[56]; Ge et al., 2015[12].)

EXCLI Journal 2017;16:602-608 – ISSN 1611-2156
Received: March 05, 2017, accepted: April 11, 2017, published: April 27, 2017

| Key findings | Reference |
|---|---|
| Resveratrol helped to improve blood supply to the femoral head in a rabbit model of osteonecrosis, which might help to protect the vascular endothelium and reduce thrombosis through an anti-inflammatory effect. | Zhai et al., 2016 |
| Dietary intake or therapeutic administration of resveratrol affects the skeletal muscle phenotype in a muscle-specific manner. Therefore, resveratrol supplementation could be a vital approach for promoting the generation of fatigue-resistant myosin heavy chain (type I) isoform, specifically if its expression is suppressed as a result of a long-term high-fat or sugar diet. | Hyatt et al., 2016 |
| The findings of the study emphasize new mechanistic insights that dihydro-resveratrol can protect against oxidative damage in the pancreas. In addition, the findings support the potential of dihydro-resveratrol for the remedy of acute pancreatitis, especially in patients who are not responsive to trans-resveratrol because they lack the necessary microbiota strains. | Tsang et al., 2016 |
| Resveratrol chemosensitized cancer cells to doxorubicin by protecting against cell proliferation and breast cancer. Additionally, co-treatment with resveratrol and doxorubicin induced apoptosis through the suppression of chronic inflammation and autophagy. | Rai et al., 2016 |
| Resveratrol influences the differentiation of preadipocytes by hindering insulin signaling, mitochondrial biogenesis, lipogenesis, and lipid storage, which contribute to weight loss in both humans and animals. | Li et al., 2016a |
| Resveratrol has beneficial effects on redox balance in bovine mammary epithelial cells. Therefore, it could be a vital therapeutic agent against oxidative insults in lactating animals. | Jin et al., 2016 |
| Resveratrol has the ability to ameliorate sporadic hypoxia-induced anxiety. Furthermore, it causes spatial learning deficits through its effects on hippocampal oxidative pathways, which may directly involve decreasing the expression of the p47Phox subunit of nicotinamide adenine dinucleotide phosphate (NADPH) oxidase. In addition, resveratrol has a potential therapeutic role in obstructive sleep apnea, which should be investigated in further studies. | Abdel-Wahab et al., 2016 |
| Resveratrol acts as a blocker of signal transduction, as well as an activator of the transcription of STAT3 and STAT5. Moreover, through these mechanisms, resveratrol may exert a potential growth inhibitory effect on renal carcinoma cells. | Kim et al., 2016 |
| Combined therapy using resveratrol and an enriched environment evidently improved the neuroprotective effect of resveratrol, compared to monotherapy using either treatment. Therefore, this combination therapy could be researched for treating ischemic brain injury. | Su et al., 2016 |
| It was reported that resveratrol showed a potent immune-enhancing activity in immunosuppressed mice. The authors indicated that the possible mechanism underlying the observed action was the activation of nuclear factor-kappa B. | Lai et al., 2016 |
| Resveratrol influenced the behavior of rats undergoing cocaine withdrawal. In addition, it was indicated that oxidative stress, inflammation, apoptosis, and silent information regulator 1 signaling pathways in the hippocampus, prefrontal cortex, or in both might be involved in mediating the observed effects of resveratrol. | Hu et al., 2016 |
| Resveratrol modified dendritic morphology in the prefrontal cortex, hippocampus, and dentate gyrus, which explained the potential therapeutic effect of resveratrol in both aging and Alzheimer's disease (AD). | Monserrat Hernández-Hernández et al., 2016 |
| Resveratrol advanced the vasoprotective effect of captopril on aortic remodeling and fibrosis during renovascular hypertension. This effect was mediated through the synergetic antioxidant and nitric oxide generation actions of resveratrol. | Natalin et al., 2016 |
| Resveratrol showed an anti-obesity effect through the induction of cytotoxicity when it was used at a high dosage. However, at lower concentrations, it influenced preadipocyte differentiation and lipolysis in mature adipocytes. | Chang et al., 2016 |
| Low-intensity exercise training combined with supplementation with resveratrol and piperine increased the mitochondrial capacity of the forearm skeletal muscle. | Polley et al., 2016 |

| Key findings | Reference |
|---|---|
| Co-treatment with diclofenac and resveratrol may represent a novel approach to reducing the dosage and gastrointestinal side effects of diclofenac. | Bedada et al., 2016 |
| Metabolic and cardiac functions improved independence of sex through dietary supplementation with resveratrol in Sprague-Dawley rats. This study showed the therapeutic role of resveratrol in the male and female rat offspring that experienced prenatal hypoxia. | Shah et al., 2016 |
| Resveratrol had beneficial effects in the liver by attenuating oxidative stress and downregulating the expression of receptor for advanced glycation end products. | Khazaei et al., 2016 |
| Resveratrol played a positive role in suppressing pancreatic cancer progression induced by hypoxia-driven reactive oxygen species (ROS) by inhibiting the hedgehog signaling pathway. This result suggests that resveratrol may be a potential candidate for the chemoprevention of cancer. | Li et al., 2016b |
| It was demonstrated that resveratrol serves as a clinically and economically feasible therapeutic agent for reducing the global burden of iron-overload cardiomyopathy at its early and chronic stages. | Das et al., 2015 |
| Resveratrol induced the apoptosis of human leukemic K562 cells via the mitochondrial signaling pathway. This finding necessitates in vivo studies on the potential use of resveratrol as an anticancer agent. | Wang et al., 2015 |
| It was demonstrated that resveratrol is effective against ischemia-induced apoptosis in the rat hippocampus through its neuroprotective and antioxidant effects. | Meng et al., 2015 |
| Resveratrol inhibited glucose uptake and had a significant antineoplastic effect in a preclinical mouse model of ovarian cancer. In addition, treatment with resveratrol suppressed tumor regrowth after therapy with cisplatin, suggesting that resveratrol has the potential to prolong disease-free survival. | Tan et al., 2016 |
| Resveratrol exerted both anti-apoptotic and anti-catabolic effects on compression injury in the skeletal muscle of rats. However, these effects required the action of silent mating type information of 2 homolog 1. | Sin et al., 2015 |
| The authors demonstrated the potential therapeutic use of resveratrol to prevent the development of proliferative vitreoretinopathy by targeting retinal pigment epithelial cells during their epithelial to mesenchymal transition. | Ishikawa et al., 2015 |
| It was shown that resveratrol has a considerable antiepileptic effect. In addition, it has a neuroprotective effect and counters epileptic depression. The findings indicated that resveratrol has the potential to be a new antiepileptic drug; therefore, more studies are needed to further elucidate its therapeutic potential. | Lu and Wang, 2015 |
| Resveratrol alleviates hepatic steatosis and inflammation in choline-deficiency-induced non-alcoholic steatohepatitis (NASH) through autophagy. Therefore, resveratrol may be useful for inhibiting accumulation of lipids and the inflammatory processes associated with NASH. | Ji et al., 2015 |
| It was reported that habitual dietary intake of resveratrol is associated with a low risk of developing frailty syndrome in community-dwelling elderly people. This effect was observed during the first 3 years of follow-up but not after longer follow-up periods. | Rabassa et al., 2015 |
| Intake of fructose for a long period may have different metabolic and vascular effects on male and female rats; however, these effects were observed to be modified by resveratrol. | Pektaş et al., 2015 |
| It was documented that resveratrol is a compound for the potential treatment of thrombovascular diseases through its antiplatelet effect. This effect was observed to occur via the inhibition of ROS production induced by NADPH oxidase and the subsequent oxidative inactivation of SHP-2. | Jang et al., 2015 |
| This recent study provided direct electrophysiological evidence of the inhibitory effect of resveratrol on pyramidal neurons. This effect was mediated, at least in part, by the reduction of evoked neural activity. | Meftahi et al., 2015 |
| It was demonstrated that attenuation of inflammatory responses might be involved in the mechanisms underlying the cardioprotective effect of resveratrol. The findings showed the response of resveratrol to myocardial ischemia/reperfusion injury. | Dong et al., 2015 |

| Key findings | Reference |
|---|---|
| Resveratrol improved the hepatic and peripheral insulin resistance induced by free fatty acids. Therefore, it may help to mitigate the health consequences of obesity. | Pereira et al., 2015 |
| The genotoxic potential of resveratrol might be attributed to its sulfate- and glucuronide-phase II metabolites through the inhibition of topoisomerase II activity. In addition, resveratrol-3-sulfate might serve as a pool for the parent compound by deconjugation at its target site. | Schroeter et al., 2015 |
| Diseases involving pathological neovascularization could be managed based on the therapeutic value of resveratrol. | Lee et al., 2015 |
| Resveratrol modified kidney histology and leptin expression level in diabetic rats through its antioxidant and antidiabetic effects. Therefore, resveratrol might be useful in the prevention of kidney damage caused by long-term hyperglycemia. | Yaylali et al., 2015 |
| Resveratrol performed more favorably than famotidine did because resveratrol prevents long-term methotrexate toxicity but does not inhibit gastric acid secretion. | Arslan et al., 2015 |
| Resveratrol exhibited a therapeutic potential against myeloproliferative neoplasms, which is evident of its aberrant activation of the janus kinase 2 pathway. | Trung et al., 2015 |
| Resveratrol emerged as a potential agent for the treatment of conditions associated with androgen excess, such as polycystic ovarian syndrome. However, further studies are required to evaluate the efficacy of resveratrol for the treatment of gynecological conditions. | Ortega and Duleba, 2015 |
| Resveratrol was recognized as a safe compound since no significant toxic effects were observed after several concentrations were tested. In addition, it was considered as an effective anti-atherogenic agent. | Riccioni et al., 2015 |
| Resveratrol was found to be a potential agent for reducing human rhinovirus replication and virus-induced cytokine/chemokine production. | Mastromarino et al., 2015 |
| It was considered that NADPH oxidase might be a potential target in AD treatment and that resveratrol may be used as a natural product with a therapeutic potential against AD. | Yao et al., 2015 |
| From histopathological and immunohistochemical analyses that were performed, it was observed that resveratrol ameliorates cisplatin-induced oxidative injury in the rabbit kidney. | Cigremis et al., 2015 |
| Resveratrol might be considered a potential natural product for development as a medicine or dietary supplement for the prevention and treatment osteoporosis. | Zhao et al., 2015 |
| Resveratrol ameliorated spatial learning and memory deficit in a rat model of subclinical hypothyroidism. The mechanism involved in this effect might modify the hyperactive hypothalamic-pituitary-thyroid axis and upregulate the hippocampal hypoexpression of synaptotagmin 1 and brain-derived neurotrophic factor. | Ge et al., 2015 |

## *Conflict of interest*

The authors declare no conflict of interest.

## Notes

Jae Kwang Kim and Sang Un Park (Division of Life Sciences and Convergence Research Center for Insect Vectors, Incheon National University, Incheon 22012, Korea; Phone: +82-42-821-5730, E-mail: supark@cnu.ac.kr) contributed equally as corresponding authors.

## Acknowledgements

This research was supported by Agriculture, Food and Rural Affairs Research Center Support Program, Ministry of Agriculture, Food and Rural Affairs.

## Conflict of interest

The authors declare no conflict of interest.

## Figures and Tables

**Table 1:**
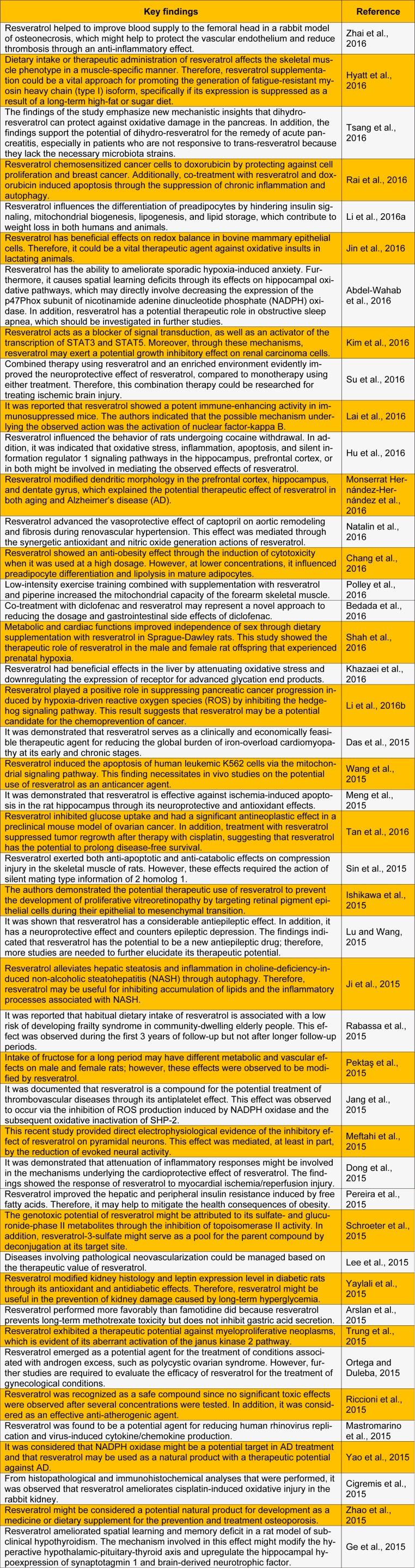
The biological and pharmacological properties of resveratrol reported by recent studies
